# Establishment of a New Murine Elastase-Induced Aneurysm Model Combined with Transplantation

**DOI:** 10.1371/journal.pone.0102648

**Published:** 2014-07-28

**Authors:** Zuzanna Rowinska, Simone Gorressen, Marc W. Merx, Thomas A. Koeppel, Elisa A. Liehn, Alma Zernecke

**Affiliations:** 1 Department of Vascular and Endovascular Surgery, Düsseldorf University Hospital, Düsseldorf, Germany; 2 Institute of Molecular Cardiovascular Research, University Hospital, RWTH Aachen University Department of Medicine, Aachen, Germany; 3 Division of Cardiology, Pneumology and Angiology, Düsseldorf University Hospital, Düsseldorf, Germany; 4 Department of Cardiology, Vascular Medicine and Intensive Care Medicine, Robert Koch Krankenhaus, Klinikum Region Hannover, Hannover, Germany; 5 Division of Vascular and Endovascular Surgery, Ludwig-Maximilian-University of Munich, Munich, Germany; 6 Institute of Clinical Biochemistry and Pathobiochemistry, University Hospital Würzburg, Würzburg, Germany; University of Amsterdam Academic Medical Center, Netherlands

## Abstract

**Introduction:**

The aim of our study was to develop a reproducible murine model of elastase-induced aneurysm formation combined with aortic transplantation.

**Methods:**

Adult male mice (n = 6–9 per group) underwent infrarenal, orthotopic transplantation of the aorta treated with elastase or left untreated. Subsequently, both groups of mice were monitored by ultrasound until 7 weeks after grafting.

**Results:**

Mice receiving an elastase-pretreated aorta developed aneurysms and exhibited a significantly increased diastolic vessel diameter compared to control grafted mice at 7 week after surgery (1.11±0.10 mm *vs*. 0.75±0.03 mm; p≤0,001). Histopathological examination revealed disruption of medial elastin, an increase in collagen content and smooth muscle cells, and neointima formation in aneurysm grafts.

**Conclusions:**

We developed a reproducible murine model of elastase-induced aneurysm combined with aortic transplantation. This model may be suitable to investigate aneurysm-specific inflammatory processes and for use in gene-targeted animals.

## Introduction

About 2–3% of people over the age of 60 are affected by an aortic aneurysm (AA), defined as an aortic dilatation of more than 1.5 times of the normal aortic diameter [1]. In 35% of all cases, these are located in the thoracic aorta, 65% affect the abdominal aorta. It is estimated that approximately 1% of patients carrying an AA die of a rupture [2]. A significant increase in the size of the aneurysm (diameter >5.5 cm) dramatically increases the risk of rupture and is associated with very high mortality rates (80%–90%) [3].

Predisposing risk factors are similar to those of atherosclerosis: male gender, age, smoking, familiar predisposition, hypertension, and hypercholesterolemia. Histologically, AA are predominated by a loss of smooth muscle cells, the destruction of elastin and collagen fibers, and an infiltration of the vessel wall with inflammatory cells [4], [5]. An aneurysmal degeneration can also occur as a complication after vascular surgery, often occurring in allografts that had previously been cryopreserved [6].

Little is known about the molecular pathways leading to AA formation. The development of syngeneic murine arterial graft models would allow to study the processes within the vessel wall during AA formation, and to assess the involvement of individual signaling molecules in vascular grafts versus systemic factors (e.g. peripheral leukocytes) when employing knockout or transgenic mouse models [7]. We here established a reproducible new mouse model that combines elastase-induced aneurysm formation [8] with aortic transplantation using the sleeve technique [9]. Our model, based on this unique combination, makes possible to describe an influence of donor and recipient cells of the genetically changed mice.

## Methods

Procedures involving animal subjects have been approved by the Institutional Animal Care and Use Committee (IACUC) at RWTH Aachen University (AZ 50.203.2-AC 26,54/06; AZ 84-02.04.2012.A234).

### Graft transplantation of the aorta treated with elastase

**Figure 1:**
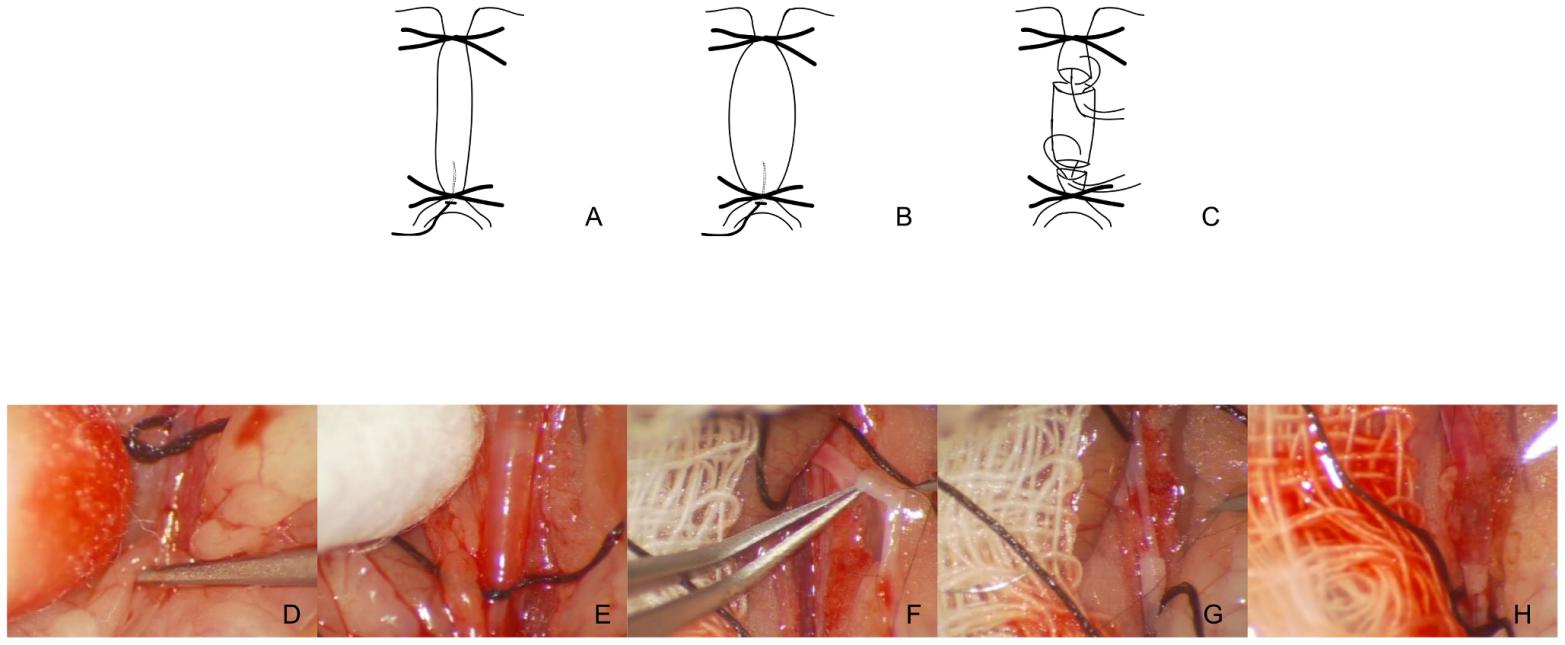
Schematic presentation of the main steps of the procedure. (A, D) Step 1: Mouse jugular catheter introduced through an aortotomy and secured with a silk tie. (B, E) Step 2: Aorta filled with saline containing type I porcine pancreatic elastase. (C, F, G) Step 3: Aortic transplantation using sleeve technique/F: Proximal anastomosis, G: Distal anastomosis/. (H) Aorta after transplantation.

C57BL/6 mice were obtained from Charles-River, Germany. Donor and recipient mice were of the same age (8–12 weeks). Donor mice were anesthetized with a mixture of 1.5 vol. % isoflurane and 100% oxygen via a facemask and laid on a platform in the supine position with all legs taped to the operating table. All hair was removed from the abdomen. An operating microscope (M650, 308 Leica Microsystems, Wetzlar, Germany) with 25× magnification was used for the procedure. Operations were performed under sterile conditions. At first, the donor underwent perfusion with elastase as described by Pyo *et al*. [8] (Figure 1). To remove the donor aorta, a mid-line abdominal incision was made and the bowel was retracted to the right. The segment of aorta between renal arteries and its bifurcation was separated from the vena cava. All small branches of this segment were secured. Temporary silk ligatures 6-0 (Silk, Deknatel, Research Triangle Park NC, USA) were placed around the proximal and distal portions of the aorta, and an aortotomy was created at the bifurcation with a 30-gauge needle. A polyurethane mouse jugular catheter (Alzet Specialized Catheters, Charles River, Germany) was introduced through an aortotomy and secured with a silk tie 6-0. Using a syringe pump calibrated to 100 mmHg, the aorta was filled with saline containing 0.414 U/ml Type I porcine pancreatic elastase (≥4.0 units/mg protein, E1250 Sigma-Aldrich, Germany). The aorta typically dilated by about 50–70% during the 5-minute period of elastase perfusion, regardless of the solution instilled. Before removing the perfusion catheter, 0.5 ml of saline solution containing 50 U of heparin was injected into the inferior vena cava. After that the catheter and the perfused segment of aorta was removed. To prepare the recipient mouse, a mid-line incision was made from the xiphoid to the pelvis, and the abdominal walls were retracted. The bowel was wrapped in saline-solution–moistened gauze and displaced to the animal's right. The infrarenal aorta was dissected free and mobilized between the renal arteries proximally and distally from the bifurcation. All small branches of this segment were secured. The proximal and distal portions of the aorta were clamped by 6-0 single silk suture (Silk, Deknatel, Research Triangle Park NC, USA). The aorta was separated between the clamps, and the cut ends were irrigated with heparinized saline to flush the lumen open. The graft was placed in the orthotopic position. The anastomosis was performed using the sleeve technique with sutures 11-0 monofilament (Ethilon, Ethicon, Norderstedt, Germany) as previously described by Dambrin *et al.* [9] (Figure 1). With this method, the end of the feeding vessel was placed into the receiving vessel. The end of the infrarenal recipient aorta was placed within the proximal end of the donor aorta. The distal anastomosis was performed in the same way. The distal end of the donor aorta was placed within the distal end of the recipient aorta. Care was taken to avoid any torsion of the aorta by the perfect alignment of donor and recipient aorta. The abdominal contents were returned to the abdominal cavity, and the wound was closed with a running 3-0 polyglycolic acid suture (Serafit, Serag-Wiessner, Naila, Germany). Control operated animals were subjected to the transplantation of the aorta without treatment with elastase. All animal protocols were approved by the local authorities and complied with animal protection law.

### High resolution ultrasound microimaging

Abdominal images were acquired applying a commercially available Visual Sonics Vevo 770 high-resolution ultrasound micro imaging system (Visual Sonics Inc., Toronto, Ontario, Canada) connected to a 30 MHz real-time microvisualization scan head. The ultrasound was performed under slight mask anesthesia by an inhaled mixture of 1.5 vol% isoflurane and 100% oxygen. Electrocardiograms (ECGs) of the animals were obtained using built-in ECG electrode-contact pads and the body temperature was maintained at 37°C using a heating pad. Heart rate and respiratory rate were controlled and maintained at 400–500 beats per minute and 100 breaths per minute, respectively. All hair was removed from the abdomen with a chemical hair remover (Nair; Carter-Horner, Mississauga, Ontario, Canada). Aquasonic 100 gel (Parker Laboratories, Fairfield, NJ, USA) was applied to the abdominal surface to optimize the visibility of the abdominal aorta. The function of the abdominal aorta and of the graft was assessed by longitudinal M-mode recording at three defined anatomic levels in elastase treated and control transplanted mice. The first level was approximately at the ostia of the renal arteries (proximal anastomosis), the second level was exactly in the middle of the inserted graft, and the third level proximal to the aortic bifurcation (distal anastomosis). At all levels, M-mode recordings were analyzed for diameter dimension, lumen dimension, anterior and posterior wall thickness as well as anterior and posterior wall displacement ratio. For this analysis the system software of Vevo 770 was applied. The imaging was performed post-operatively after 4 weeks and 7 weeks in both groups.

### Immunohistochemistry

For tissue harvesting, animals were anesthetized (with a mixture of 1.5 vol. % isoflurane and 100% oxygen via a face mask) and vessels were flushed with phosphate-buffered saline (PBS) followed by 4% formaldehyde/PBS (pH 7.4) by cardiac puncture. Subsequently, grafts were gently removed. After overnight fixation in 4% formaldehyde/PBS, specimens were further processed and embedded in paraffin. Serial sections (5 µm) of grafts of transplanted animals distal to the anastomosis site were stained by elastica-van-Gieson staining (EVG), sirius red staining (SIR), cholinesterase staining (CHE). Immunohistochemistry was performed using antibodies raised against macrophages (Mac2, CL8942AP/Cedarlane), and vascular smooth muscle cells (SMA, 1A4/Daco), detected by secondary anti-rat FITC (Jackson Immuno- Research).

### Statistics

Data are presented as mean ± SEM. The significance of changes between experimental groups at different time points was determined by 2-way ANOVA (GraphPad Prism 5.01). A p-value of <0.05 was considered significant.

## Results

### Surgical procedure and follow up

The aorta was left untreated (controls) or was perfused with elastase in donor mice as previously described [8] (Figure 1). Recipient male mice (n = 6–9) underwent infrarenal, orthotopic transplantation of the control or elastase-treated aorta using the sleeve-technique. Subsequently, mice were monitored by ultrasound examination using a Vevo 770 until 7 weeks after grafting and sacrifice of the mice in order to asses vessel wall diameter and function. All animals survived the surgical procedure and lived until sacrifice. The mean time for completion of the two aortic anastomoses was 22 minutes. A single ultrasound session ranged from 20–30 minutes per mouse.

### Ultrasound

**Figure 2:**
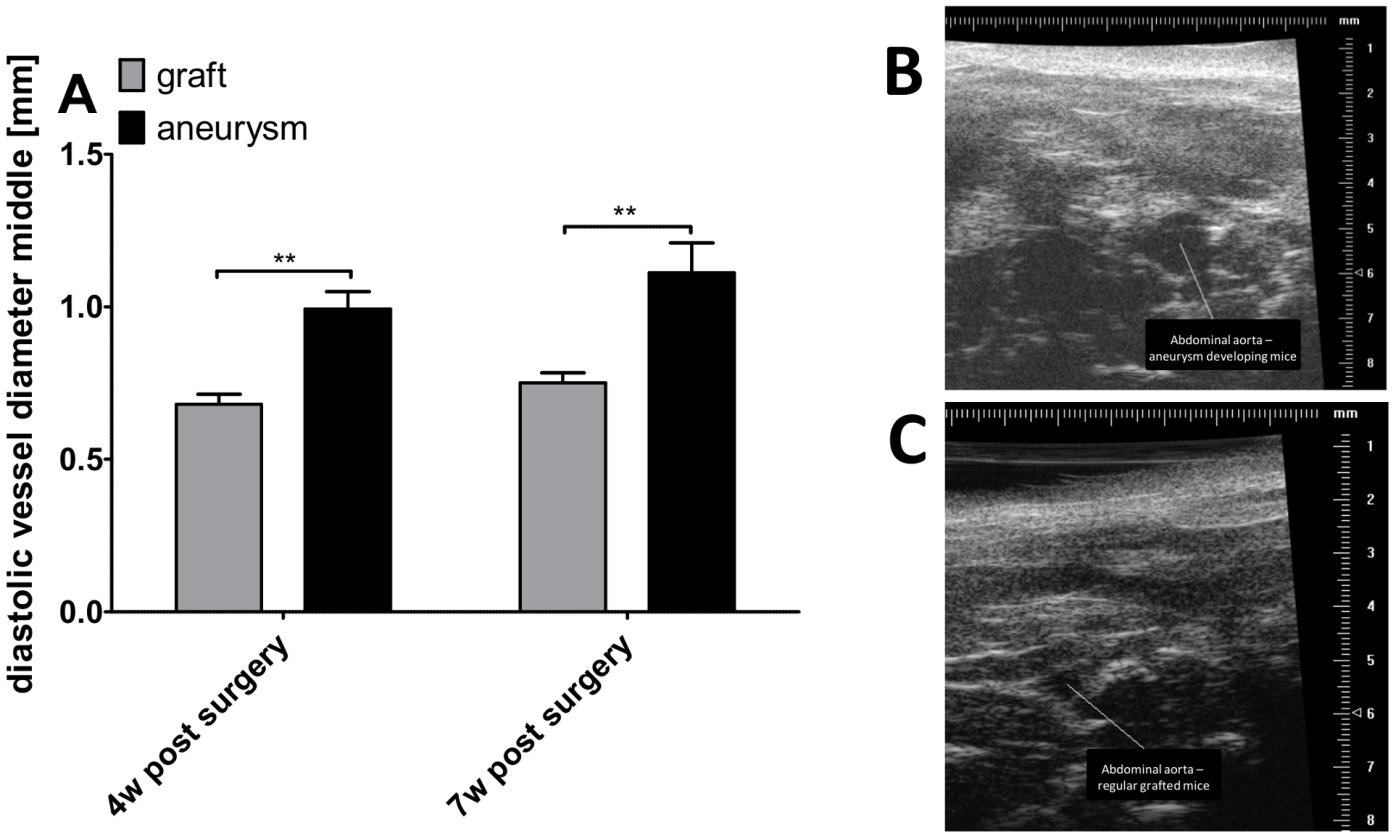
Diastolic vessel diameter assessed by high-resolution ultrasound. (A) Diastolic vessel diameters were measured in control grafted mice and mice transplanted with elastase-pretreated aortae at 4 and 7 weeks after surgery using high resolution ultrasound. (B) Representative ultrasound measurement of an aneurysm in the elastase-pretreated aortae. (C) Representative ultrasound measurement of a control grafted mouse. **p<0,01.

Transabdominal ultrasound has previously been applied to detect and monitor patency, morphology, and function of the abdominal aorta in mice. We here used a high frequency ultrasound micro-imaging protocol to access the dimensions and dynamics of the grafted vessel, as previously described by us [10]. Diastolic vessel diameter was assessed in the middle of the graft at 4 and 7 weeks after surgery. Elastase-perfused grafts exhibited a significant increase in diastolic vessel diameter compared to control-grafted mice at 4 weeks (0.99±0.06 mm *vs*. 0.68±0.03 mm; n = 7–9 mice per group; p<0.01; Table 1) and 7 week after surgery (1.11±0.10 mm *vs*. 0.75±0.03 mm; n = 6–9 mice per group; p<0.001, Figure 2 and Table 1), confirming the development of an AA in aortae pretreated with elastase.

**Table 1:** Parameters measured by high resolution ultrasound.

		Control treated aortae		Elastase treated aortae	
		4w post surgery (n = 8)	7w post surgery (n = 7)	4w post surgery (n = 8)	7w post surgery (n = 9)
Parameter	unit	mean ± SEM	mean ± SEM	mean ± SEM	mean ± SEM
**diastolic lumen proximal**	mm	0,75±0,04	0,75±0,06	0,68±0,04	0,61±0,02*
**diastolic lumen middle**	mm	0,54±0,03	0,59±0,03	0,82±0,06**	0,93±0,10**
**diastolic lumen distal**	mm	0,52±0,04	0,50±0,03	0,54±0,06	0,71±0,04**
**diastolic vessel diameter proximal (Dd)**	mm	0,93±0,04	0,92±0,06	0,85±0,05	0,78±0,02
**diastolic vessel diameter middle (Dd)**	mm	0,68±0,03	0,75±0,03	0,99±0,06**	1,11±0,10**
**diastolic vessel diameter distal (Dd)**	mm	0,69±0,04	0,67±0,03	0,68±0,06	0,88±0,05**
**anterior wall displacement proximal (δa)**	mm	0,10±0,01	0,09±0,01	0,08±0,01	0,06±0,01
**anterior wall displacement middle(δa)**	mm	0,04±0,01	0,05±0,01	0,02±0,00	0,03±0,00*
**anterior wall displacement distal(δa)**	mm	0,02±0,00	0,04±0,01	0,02±0,01	0,02±0,01
**posterior wall displacement proximal(δp)**	mm	0,05±0,01	0,04±0,01	0,04±0,01	0,02±0,00
**posterior wall displacement middle (δp)**	mm	0,02±0,01	0,03±0,01	0,02±0,01	0,02±0,00
**posterior wall displacement distal (δp)**	mm	0,03±0,01	0,02±0,00	0,02±0,01	0,01±0,01
**anterior wall thickness proximal**	mm	0,09±0,00	0,09±0,01	0,08±0,01	0,08±0,01
**anterior wall thickness middle**	mm	0,07±0,01	0,08±0,00	0,08±0,01	0,10±0,01
**anterior wall thickness distal**	mm	0,08±0,00	0,08±0,00	0,07±0,01	0,07±0,00
**posterior wall thickness proximal**	mm	0,10±0,00	0,09±0,01	0,09±0,01	0,08±0,00
**posterior wall thickness middle**	mm	0,07±0,00	0,08±0,00	0,09±0,01	0,09±0,01
**posterior wall thickness distal**	mm	0,08±0,00	0,09±0,00	0,08±0,01	0,07±0,00**
**δa/Dd anterior wall displacement ratio proximal**		0,10±0,01	0,09±0,00	0,09±0,01	0,07±0,01
**δa/Dd anterior wall displacement ratio middle**		0,05±0,01	0,07±0,01	0,03±0,00*	0,03±0,01**
**δa/Dd anterior wall displacement ratio distal**		0,03±0,00	0,05±0,01	0,02±0,00	0,03±0,00
**δp/Dd posterior wall displacement ratio proximal**		0,05±0,00	0,04±0,00	0,04±0,00	0,03±0,00
**δp/Dd posterior wall displacement ratio middle**		0,03±0,01	0,04±0,01	0,02±0,01	0,01±0,00*
**δp/Dd posterior wall displacement ratio distal**		0,03±0,00	0,02±0,00	0,01±0,01	0,01±0,00

Indicated parameters were measured in control grafted mice and mice transplanted with elastase-pretreated aortae at 4 and 7 weeks after surgery. * p≤0,05; ** p ≤0,01; *** p ≤0,001 *versus* controls.

**Figure 3:**
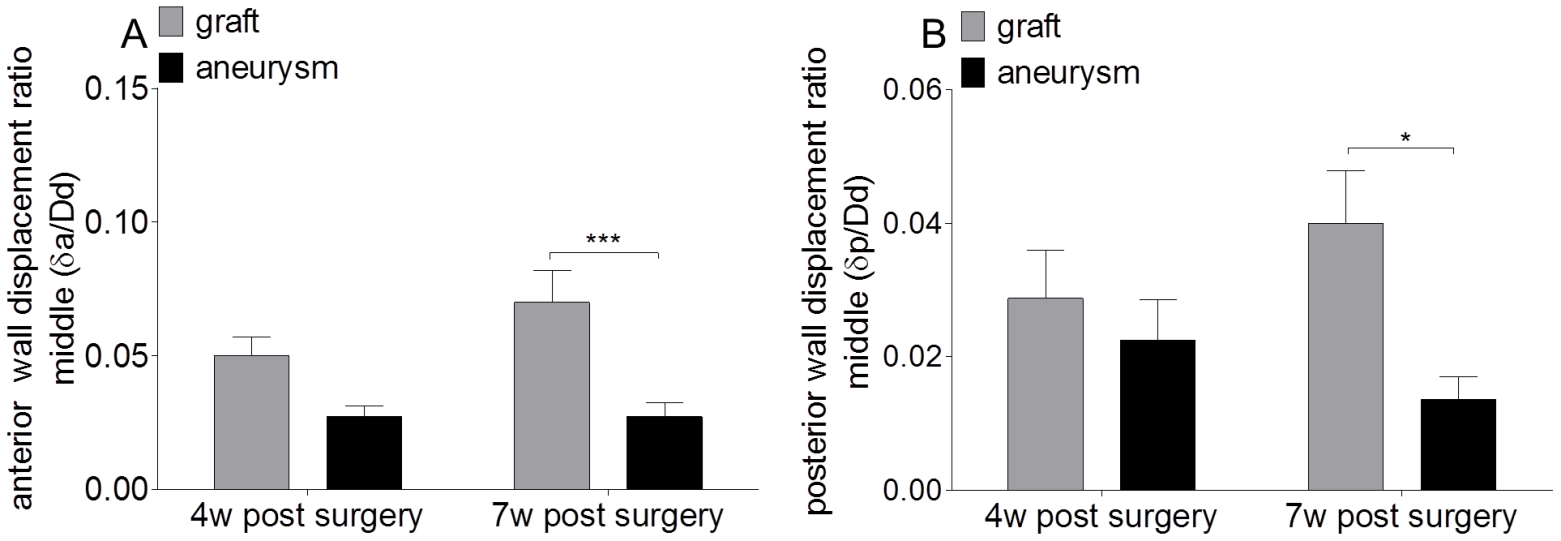
Anterior δa/Dd and posterior δp/Dd wall displacement ratio. (A) Anterior and (B) posterior wall displacement ratios were measured in control grafted mice and mice transplanted with elastase-pretreated aortae at 4 and 7 weeks after surgery using high resolution ultrasound. *p<0,05, ***p<0,001.

We further measured the anterior and posterior wall displacement ratio (δa/Dd) in control and elastase-perfused grafts in order to compare wall motion between the groups. Aneurysmatic, elastase-perfused grafts displayed a slightly decreased anterior wall displacement ratio at 4 weeks (0.05±0.01 mm *vs*. 0.03±0.00 mm; n = 8 mice per group; Table 1) and significantly decreased anterior wall displacement ratio at 7 weeks after surgery (0.07±0.01 mm *vs*. 0.03±0.01 mm; n = 7–9 mice per group; p<0.001; Table 1) compared to control-grafted mice. Furthermore, a decreased posterior wall displacement was observed in elastase-treated grafts at 7 weeks after surgery compared to controls (0.04±0.01 mm *vs*. 0.01±0.00 mm; n = 7–9 mice per group; p<0.05, Figure 3 and Table 1). This indicates that aneurysm formation was associated with a diminished anterior and posterior wall motion at 7 weeks after surgery compared to control-grafted mice.

### Histopathology and immunohistochemical analysis (IHC)

**Figure 4:**
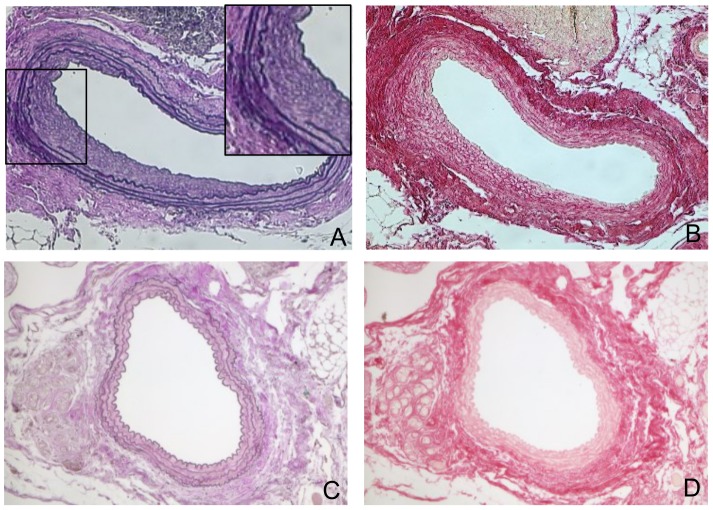
Representative examples of histological cross-section (EVG, SIR). A,B) Elastase treated aortae after 7 weeks: (A) Elastic van Gieson (EVG) staining (×100, ×200) reveals destruction of medial elastin layer, neointima formation, but no existence of pseudoaneurysms. (B) Sirius Red Staining (SIR) (x100) shows substantial collagen content. C,D) Control grafted aortae after 7 weeks: (C) Elastic van Gieson (EVG) staining (x100) shows no destruction of the medial elastin layer, no existence of aneurysms or no neoitimal formation, (D) Sirius Red Staining (SIR) (x100) shows only low collagen content.

**Figure 5:**
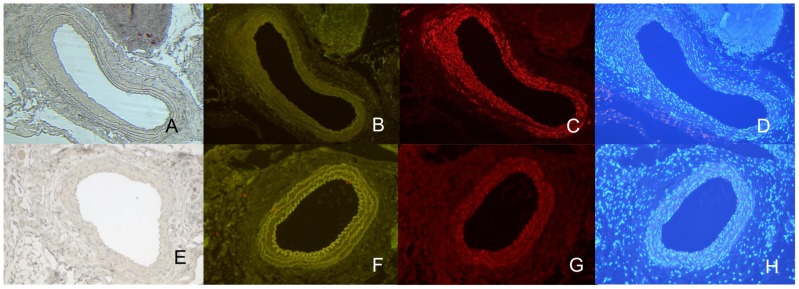
Representative examples of histological and immunohistochemical cross-section (CHE, MAC2, SMA). A–D) Elastase treated aortae after 7 weeks: (A) Cholinesterase (CHE) staining (x100) shows absence of neutrophils, (B) MAC2 (x100) staining shows absence of macrophages, (C) alpha-Smooth Muscle Actin (SMA) staining (x100) shows abundant content of smooth muscle cells, (D) DAPI staining. E–H) Control grafted aortae after 7 weeks: (E) Cholinesterase (CHE) staining (x100) shows absence of neutrophils (F), MAC2 (x100) staining shows no accumulation of macrophages, (G) alpha-Smooth Muscle Actin (SMA) staining (x100) shows minimal loss of smooth muscle cell actin, (H) DAPI staining.

To furthermore assess the vessel-wall morphology of the grafts, histological analyses were performed after 7 weeks of surgery. In sections through both the elastase-pretreated graft and controls no pseudo-aneurysms were detectable. However, moderate intimal hyperplasia could be evidenced within grafts. Moreover, disruption of medial elastin could be detected in elastase-pretreated aortae but not control vessels at sites of neointimal hyperplasia. Immunofluorescence staining demonstrated that the media and areas of intimal hyperplasia were predominantly populated by smooth muscle cells (SMA). Accordingly, an increased content of collagen (Sirius Red staining) was observed in elastase-pretreated grafts, whereas only few macrophages (Mac2) and no neutrophils (cholinesterase) could be evidenced in the intima of the aneurysm group (Figure 4 and 5).

## Discussion

Various aneurysm models have been established in mice in recent years. Among these, the elastase-induced aneurysm model is the most widely used animal model of abdominal AA (AAA) since first described in rats by Anidjar *et al.* [11]. This model, which locally applies elastase, causes AAA of smaller diameter in the infrarenal aorta in contrast to larger suprarenal aneurysms induced by systemic Angiontensin II-infusion [12], [13].

In addition, pharmacological interventions can be initiated directly after transplantation during the formation of the aneurysm or also later, because the dilatation is also present between 4 and 7 weeks. In the AngII model, the interventions usually start 1 week after AAA induction and aneurysms are analyzed at week 4.

The elastase-induced model of AAA has successfully been used to examine the role of chronic inflammation and specific matrix-degrading enzymes as well as of different pharmacological agents or genetic alterations in aortic degeneration and aneurysm formation [14].

Previously, two other models using transplantation have been described to study specific molecular mechanisms leading to aneurysm formation. Shimizu *et al*. transplanted aortic allografts into Interferon (IFN)-γ receptor-deficient mice as an immunologically driven model of aneurysm formation to test the hypothesis that skewing towards a Th2-enriched environment induces abdominal aorta aneurysm (AAA) formation. Thus, the Shimizu model is based on using genetically engineering mice: after aortic transplantation, allografts in IFN-γ receptor-deficient hosts developed aortic aneurysms, but not WT mice. This model can therefore only be used in the IFN-γ receptor-deficient background, but is not applicable in other strains, and therefore lacks transferability [15].

Goldberg *et al*. induced aneurysms in mice receiving an aortic transplant with injections of a pro-inflammatory viral serpin with a mutated reactive site [16], primarily interested in the ensuing acute inflammatory response.

We here established a new and highly reproducible model of AA formation, combined with aortic transplantation. Aortic transplantation was performed using the sleeve-technique. As recently described by our group, this technique results in reliable anastomoses compared to conventional end-to-end suture methods [9] and *per se* does not impair vessel morphology or function [10]. In this model, the acute inflammatory reaction is thus low, allowing the assessment of chronic changes of the vessel wall.

Different methods have been applied to assess the dimensions of AAA. For example, in a study of Pyo *et al*. the aortic diameter was measured using a calibrated ocular grid [8]. Azuma *et al*. compared the results from ultrasound examination in the elastase-induced aneurysm model with video microscopy results. In this study, the aortic luminal diameter (ALD) was assessed in transverse image scans and cine loops of 300 frames throughout the infrarenal region of the abdominal aorta, demonstrating that high-frequency ultrasound provides reliable ALD measurements in developing murine abdominal aortic aneurysms [17]. Ultrasound was also successfully applied for assessing angiotensin-induced aneurysm model in the study by Cao *et al*. [18]. We employed high-resolution ultrasound because of its high reproducibility and the possibility to acquire not only the vessel wall dimensions, but also the dynamics of the abdominal aorta at low experimental strain for the animals. We observed a maximum mean aortic diameter of the aneurysmatically changed vessel wall of 1.11 mm, similar to findings by Bartoli *et al*. and Azuma *et al*. in C57BL/6 mice (1.15 mm and 1.25 mm) [13], [17].

As end time point, we have chosen the 7-week, because the elastase treaded aorta achieved 1.5× size of a normal aorta (increase in diameter of 1.5-fold) after that time. However, this does not exclude that after 7 weeks further dilation may occur. It is known, that the aneurysm in the elastase model does not achieve as big a diameter as in the Angiotensin II model [19], therefore it is easier to control and standardized.

Histologically, Azuma *et al*. and Saatchi *et al*. observed an increased wall thickness and neointima formation after 28 days within aneurysms [17], [20]. In our study, we similarly observed the formation of intimal hyperplasia at 7 weeks after elastase-treatment and transplantation, as well as destructive changes of the medial aortic wall, and an increased smooth muscle cell and collagen content. This phenotype increases the stiffness of the vessel and favors brakeage. In opposition with Pyo *et al*., increased collagen content may be explained through a prolonged period of remodeling in our longer term experiments [8], [17], [20]. In addition, we as well as Azuma *et al.* ^17^ and Saatchi *et al.* ^19^ did not observe a significant accumulation of macrophages, while Pyo *et al*. described that macrophages (detected by Mac3 staining) were the dominant cell type detected by immunohistochemistry [8]. This could furthermore indicate an early stage of the aneurysm, favoring the initiation of neointimal formation, and resolve at later stages.

AAA is a complex disease. A reproducible model of elastase-induced AAA combined with aortic transplantation offers the unique possibility to evaluate the differential contribution of donor/recipient cells. Moreover, our model allows that the aortic segment could be infected or transfected with viral or other constructs carrying a gene of interest, or incubated with an antagomir against a specific microRNA before transplantation in order to clearly dissect a local influence of the manipulated gens or pathway during aneurysm formation. This may be of particular interest, when studying the role of microRNAs in development of aneurysms, given the emerging evidence of the important role of non-coding RNAs in the pathogenesis of aortic aneurysm. For instance, Maegdefessel *et al.* demonstrated that modulation of miR-29b expression affects aortic aneurysm progression in two mouse models of disease [21]. The role of miR-29 in aortic aneurysm formation was also investigated by Boon *et al.*, and it was shown that its inhibition abrogated aortic dilation in mice, suggesting that miR-29 represents a novel target to augment matrix synthesis and maintain vascular wall structural integrity [22].

We therefore believe that our model will help to identify key processes that play a role in aneurysm formation.
